# Neutralizing Antibodies as Predictors of Vaccine Breakthrough Infection in Healthcare Workers Vaccinated with or without a Heterologous Booster Dose: A Cohort Study during the Third COVID-19 Wave in Peru

**DOI:** 10.3390/vaccines11020447

**Published:** 2023-02-15

**Authors:** Miguel Hueda-Zavaleta, Juan C. Gómez de la Torre, José Alonso Cáceres-DelAguila, Cecilia Muro-Rojo, Nathalia De La Cruz-Escurra, Cesar Copaja-Corzo, Carlos J. Aragón-Ayala, Vicente A. Benítes-Zapata

**Affiliations:** 1 Facultad de Ciencias de la Salud, Universidad Privada de Tacna, Tacna 23003, Peru; 2 Hospital III Daniel Alcides Carrión—Essalud Tacna, Tacna 23041, Peru; 3 Roe Clinical Laboratory, Lima 15076, Peru; 4 Facultad de Ciencias de la Salud, Universidad Científica del Sur, Lima 15842, Peru; 5 Facultad de Medicina, Universidad Nacional de San Agustin de Arequipa, Arequipa 04000, Peru; 6 Unidad de Investigación para la Generación y Síntesis de Evidencias en Salud, Universidad San Ignacio de Loyola, Lima 15024, Peru

**Keywords:** COVID-19, SARS-CoV-2, vaccine breakthrough infection, humoral immunity, heterologous booster vaccine, neutralizing antibody

## Abstract

We evaluated neutralizing antibody (NAbs) levels as a protective factor against vaccine breakthrough infection (VBI) in healthcare workers (HCWs) during the third COVID-19 wave in Peru. This retrospective cohort study employed the information from a private laboratory in Lima (Peru) of HCW who received only two BBIBP-CorV vaccines or (additionally) a heterologous booster with BNT162b2. We evaluated the association between the VBI and the levels of NAbs at 21, 90, 180, and 210 days after the BBIBP-CorV second dose. NAbs were calculated with the cPass™ SARS-CoV-2 Neutralization Antibody Detection kit (surrogate virus neutralization test (sVNT)) and the Elecsys^®^ anti-SARS-CoV-2 S Test. Of the 435 HCW evaluated, 31.72% had an infection previous to vaccination, 68.28% received a booster dose, and 23.21% had a VBI during the third wave. The variables associated with a lower risk of VBI were male sex (aRR: 0.43) and those who had (180 days after BBIBP-CorV inoculation) NAbs levels ≥ 60% (aRR: 0.58) and ≥90% (aRR: 0.59) on cPass™, and ≥500 with Elecsys^®^ (aRR: 0.58). HCW whose NAbs persisted at higher levels six months after the BBIBP-CorV showed a lower risk of suffering from a VBI during the third COVID-19 wave.

## 1. Introduction

The inactivated virus vaccine against SARS-CoV2, the BBIBP-CorV (Beijing Bio-Institute of Biological Products Co., Ltd., Beijing, China), has not only shown efficacy in a short time during clinical trials [1,2] but also in real environments [3]. However, its long-term efficiency is still unclear, it having been recognized that there is a progressive diminution of the humoral response through time [4]. Even when vaccination campaigns globally have shown promising results [5], many countries have experienced an increment in the number of clinical cases provoked by the virus (COVID-19) [6,7,8]. This phenomenon might have been caused by new subtypes of the virus such as the Omicron (B.1.1.529) variant, which the World Health Organization (WHO) classified as a variant of concern (VOC) (26 November 2021). 

This variant has shown a higher infectivity and evasion capacity than other variants of concern [6,9,10], provoked by its several mutations on the spike protein [11]. These characteristics, plus the diminishment of the humoral response, encouraged the WHO and many countries to recommend a third dose (for booster effect) against it [12]. For this purpose, on 15 October 2021, the Peruvian Ministry of Health recommended a heterologous booster dose with the vaccine BNT162b2 (Pfizer-BioNTech, New York, NY, USA; Mainz, Germany) to HCW [13].

Recent studies have shown that booster doses increase the level of neutralizing antibodies against the variants of concern, which may decrease infection rates and severe illness from COVID-19 [6,9,14,15,16]. Nevertheless, most studies only evaluated this effect with vaccines ARNm in a homologous booster schedule and did not evaluate its real-world effectiveness in heterologous booster schedules con inactivated vaccines. 

The study’s objective was to determine if the level of neutralizing antibodies (NAbs) at 21, 90, 180, and 210 days after the second dose of BBIBP-CorV has a protective factor against COVID-19 vaccine breakthrough infection (VBI) in HCW during the third wave in Peru. The third wave period occurred approximately between 4 January and 4 April 2022 [17], during which time the Omicron was the causative pathogen in more than 94% of COVID-19 cases [18].

## 2. Materials and Methods

### 2.1. Population and Study Design

We generated this retrospective cohort study with the information from a secondary data source obtained from HCW in a private clinical laboratory in Lima, Peru. The data were supplied by the laboratory’s occupational health area department, which carried out this evaluation for biosecurity purposes during the pandemic period. The HCW who voluntarily decided to participate gave informed consent. None of the participants refused this follow-up. The study protocol was approved by the Institutional Research Ethics Committee of the Faculty of Health Sciences from the Private University of Tacna on 24 March 2021.

The study’s time range was from 9 February 2021 (when the first BBIBP-CorV vaccination campaign for HCW started in Peru) [19] to 4 March 2022. We chose this last date because the rate of cases declined considerably at this time (one month before the third wave’s end), and a similar study employed this same criterion [20]. For protocolar purposes, the two BBIBP-CorV injections were separated by 21 days between doses, while the heterologous booster dose with BNT162b2 started on 15 October 2021 (a temporary separation of 6 months between doses, approximately) [21]. Another inclusion criterion for the study was to have worked in the private clinical laboratory during the study period.

According to the state of vaccination, we categorized the HCW in two groups: those vaccinated with only two BBIBP-CorV doses, and those with a heterologous combination of the BBIBP-CorV and the BNT162b2 booster dose. It is relevant to mention that the patients with the booster dose were considered until 31 December 2021, when the evaluation of infection risk based on the levels of antibodies started. 

The considerate variables were age (in years), sex, previous SARS-CoV2 infections, and time between reinfection (in days). To differentiate the HCW previously infected (PI) from those who were not (NPI), we considered any positive molecular test (RT-PCR) or antigen test before the third wave (i.e., before 4 January 2022) and their serological records before receiving the first dose. We determined their serological history by employing the Elecsys^®^ Anti-SARS-CoV-2 (Roche Diagnostics International AG, Rotkreuz, Switzerland) and the Aeskulisa SARS-CoV-2 S1 IgG y IgM (Aesku. Diagnostics GmbH & Co. KG, Wendelsheim, Germany). 

**Figure 1:**
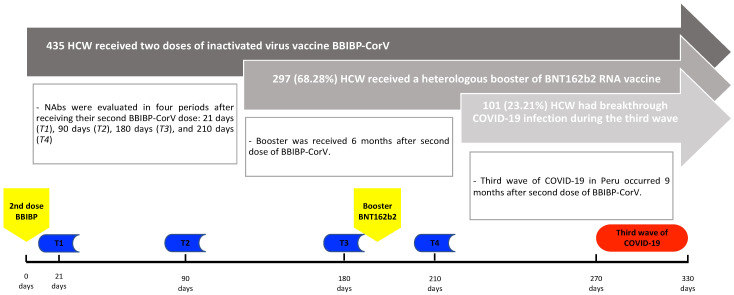
Timeline of the serological evaluations according to the vaccination and third COVID-19 wave period.

Regarding the humoral response from the HCW, this variable was evaluated in four periods after receipt of a second BBIBP-CorV dose: 21 days (T1), 90 days (T2), 180 days (T3), and 210 days (T4) after the second dose (Figure 1). The humoral response was evaluated using the Elecsys^®^ Anti-SARS-CoV-2 S (Roche Diagnostics GmbH, Mannheim, Germany) and the Neutralizing Antibodies SARS-CoV-2 cPass™ (GenScript Biotech Corporation, Piscataway, NJ, USA). Both tests were approved by the Food and Drug Administration (FDA) and have a strong correlation between them [14,22,23]. The cPass™ SARS-CoV-2 neutralizing antibody detection kit (also known as the surrogate virus neutralization test (sVNT)) has shown high sensitivity and specificity, and a strong correlation with the gold standard considers a positive result a percentage of the inhibition signal (PIS) higher than or equal to 30% [24,25]. The Elecsys^®^ is a quantitative immunoassay that detects high-affinity antibodies against the SARS-CoV-2 protein S receptor-binding domain (RBD), on which any results higher or equal than 0.8 U/mL are considered positive [26]. 

### 2.2. Outcome

We defined vaccine breakthrough infection (VBI) as a positive COVID-19 result (employing a rapid antigen test or a real-time reverse transcription-polymerase chain reaction (RT-PCR)) in any vaccinated HCW with a suspected clinical profile or exposure to COVID-19 patients (CDC, 2020). We applied this definition during our population’s study from 4 January to 4 March 2022. We did not evaluate the HCW’s samples, instead employing genomic sequencing, assuming that most of those infections were provoked by the Omicron subtype (B.1.1.529) between the first of January 2022 and the end of the study period. We based this presumption on the genomic epidemiological follow-up reported by the Nation Health Institute, which noted that this variant was predominant (94–100%) during that period in Lima, Peru [18,27]. 

### 2.3. Statistical Analysis

We analyzed the database provided by the laboratory’s occupational health area, employing the statistical program STATA v17.0 (StataCorp, College Station, TX, USA) and Prism V 9.2.0 (Graphpad Software, San Diego, CA, USA). We considered presenting the qualitative variables as absolute frequency and percentage. For the quantitative variables, we represented them with the median and interquartile range due to the asymmetric distribution of the sample. We compared the proportion and the geometric mean titers (GMT) of the NAbs of HCW with VBI and those who did not present a breakthrough infection during the third wave in Peru, employing Pearson’s chi-squared, Fisher’s exact test or the Mann–Whitney U test as appropriate. We determined the comparison between NAbs in each HCW group during the follow-up (with and without COVID-19) by employing Wilcoxon’s non-parametric sign-and-rank statistical test. A value of *p* < 0.05 was considered statistically significant. 

To establish the associated variables with the VBI, we employed the Poisson regression model with robust variance to determine the relative risk (RR), both crude and adjusted, with a 95% confidence interval (95% CI). The variables that were significant in the crude regression analysis, as well as the vaccination status and the previous history of infection, were entered into the adjusted model.

## 3. Results

**Figure 2:**
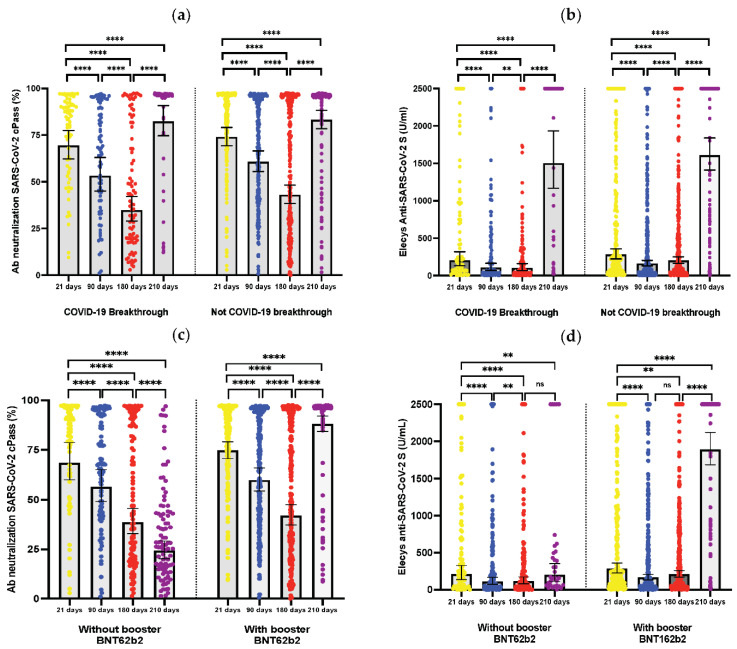

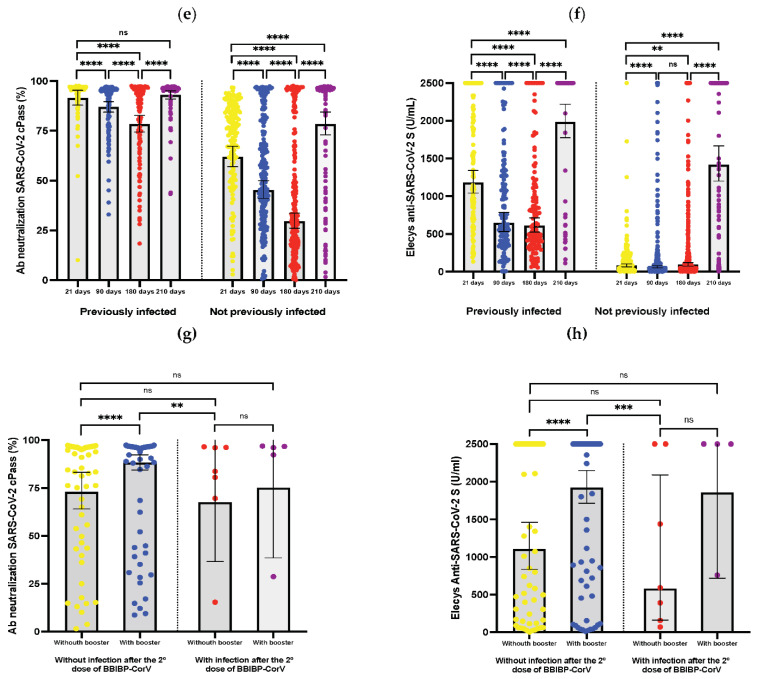
Humoral response kinetics to the inactivated SARS-CoV-2 BBIBP-CorV vaccine, determined up to 210 days after the second dose, in healthcare workers with and without vaccine breakthrough infection: (**a**) Antibody neutralization cPass™ after the second dose of BBIBP-CorV vaccination. (**b**) Titers of Elecsys^®^ anti-SARS-CoV-2 S after the second dose of BBIBP-CorV vaccination. Humoral response between healthcare workers with and without booster BNT162b2, determined up to 210 days after the second dose of BBIBP-CorV: (**c**) antibody neutralization cPass™; (**d**) titers of Elecsys^®^ anti-SARS-CoV-2 S. Humoral response between healthcare workers previously and not previously infected, determined up to 210 days after the second dose of BBIBP-CorV; (**e**) antibody neutralization cPass™; (**f**) titers of Elecsys^®^ anti-SARS-CoV-2 S. Humoral response of healthcare workers with and without a history of infection after the second dose of BBIBP-CorV with or without booster BNT162b2; (**g**) antibody neutralization cPass™ after the second dose of BBIBP-CorV vaccination; (**h**) titers of Elecsys^®^ anti-SARS-CoV-2 S after the second dose of BBIBP-CorV vaccination. ns = *p* ≥ 0.05; ** = *p* < 0.05; *** = *p* < 0.001; **** = *p* < 0.0001.

An initial group of 471 HCW fit the cohort inclusion criteria; nevertheless, we excluded 36 HCW because they stopped working in the private laboratory. A total of 435 HCW were evaluated. A total of 78.16% (*n* = 340) of the HCW were women, and the median age was 34 years old (IQR: 28–43). Some 31.72% (*n* = 138) of the HCW had a history of COVID-19 infection, the majority before the first dose of BBIBP-CorV (*n* = 122); the median time from the first infection until the third wave was 507.5 days (IQR: 317–558). Some 68.28% (*n* = 297) of the HCW received a heterologous booster dose. Finally, 23.21% (*n* = 101) of HCW showed VBI during the third wave in Peru (Figure 2), with a median time from the first infection until VBI of 555 (IQR: 366–610) in HCW with the infection before the first dose of BBIBP-CorV, and 175.5 (135–208) in HCW with infection after the second dose of BBIBP-CorV (Table 1). No HCW who were infected after the first dose of BBIBP-CorV (but before the second dose) developed vaccine breakthrough infection. 

Concerning the humoral response, the seropositivity proportion of NAbs evaluated with cPass™ decreased progressively after the second BBIBP-CorV dose, from 94.64% at 21 days after (T1) to 68.15% at 180 days after (T3), followed by an increment at 210 days (T4) of 94.78%. However, we did not observe this effect with Elecsys^®^ anti-SARS-CoV-2 S, with which it could be seen that seropositivity proportions remained high during follow-up. In the bivariate analysis, the variables statistically associated with VBI were sex (*p* = 0.02), as well as having an Elecsys^®^ anti-SARS-CoV-2 S result 180 days after (T3) that was lower than 500 (*p* = 0.008), and lower than 1000 (*p* = 0.05). Another variable associated was the level of NAbs evaluated with cPass™, on which the results 21 days after (T1) were lower than 60% (*p* = 0.04) and 180 days after (T3) were lower than 60% (*p* = 0.002) and 90% (*p* = 0.02) (Table 1).

The associated protective factors against VBI that we evaluated with Poisson regression models and a robust error variance were male sex (cRR: 0.57; 95% CI: 0.34–0.96; *p* = 0.03), NAbs with cPass™ at 21 days (T1) ≥ 60% (cRR: 0.61; 95% CI: 0.39–0.95; *p* = 0.03), NAbs with cPass™ at 180 days (T3) ≥ 60 (cRR: 0.55; 95% CI: 0.37–0. 82; *p* < 0.01) and ≥ 90% (cRR: 0.57; 95% CI: 0.35–0.93; *p* = 0.02), and NAbs evaluated with Elecsys^®^ at 180 days (T3) ≥ 500 (cRR: 0.55; 95% CI: 0.35–0.86; *p* = 0.01) (Table 2).

We observed that the variables associated with a protection effect against VBI in the crude Poisson regression analysis were also in the adjusted analysis, these being the male sex (aRR: 0.43; 95% CI: 0.22–0.81; *p* = 0.01), NAbs measured on cPass™ at 21 days (t1) ≥ 60% (aRR: 0.62; 95% CI: 0.39–0.87; *p* = 0.03), NAbs measured with cPass™ at 180 days (T3) ≥ 60% (aRR: 0.58; 95% CI: 0.39–0.87; *p* < 0.00), ≥ 90% (aRR: 0.59; 95% CI: 0.37–0.96; *p* = 0.03), and NAbs measured with Elecsys^®^ at 180 days (T3) ≥ 500 (aRR: 0.58; 95% CI: 0.37–0.91; *p* = 0.01) (Table 2).

## 4. Discussion

In this retrospective cohort study, we denoted that close to 25% of HCW who received two doses of the inactivated-virus vaccine BBIBP-CorV (with or without a booster shot with the ARNm vaccine BNT162b2) presented VBI during the third wave of COVID-19 in Peru. The variables associated with a lower risk of VBI were male sex, the presence of NAbs (examined by cPass™) 180 days after the second BBIBP-CorV dose with a PSI equal or higher than 60%, or equal or higher than 90%, and Elecsys^®^ values equal or higher than 500. The background of having received a heterologous booster with BNT162b2 at seven months after the second dose of BBIBP-CorV and a history of previous SARS-CoV-2 infection were not associated with a lower risk of VBI.

The incidence of VBI in this study was higher than in the evidence reported by other authors. One systemic study of HCW (before the expansion of Omicron) reported an incidence of 0.1% to 1% in the first six months after receiving the second BNT162b2 dose or a ARNm-1273 dose (Moderna, Cambridge, USA) [28]. Recent data from the New York Health Department reported that the prevalence of VBI was 9.9% [29]. It is relevant to note that the time that lapsed between the second vaccine dose and the VBI in our study group was 9 to 10 months. We expected a decrement in their immunity [16,30,31] and therefore a greater risk of VBI, which could explain the lower proportions of VBI in previous studies with a shorter follow-up time.

We also need to consider the capacity of Omicron for immunological evasion in those previously infected, and in those vaccinated [6,9,10,11], Omicron was the predominant variant of the virus in the third wave [18,27]. The multiple mutations on the spike protein confer a reduced response provoked previously by natural infection or immunization, which diminishes the neutralization activity by about 33 and 44 times until there is a complete loss of neutralizing capacity [32]. This is a greater diminishing than that observed with other variants such as Delta, Alpha, Beta, Gamma, and Lambda [33]. These last two were the variants prevalent in our country during the second wave [34]. Most of the HCW in our study were infected during this period. However, neither the fact of having been previously infected nor the time of this infection was associated with a risk of VBI in our study.

We did not find differences when comparing the VBI proportion of the group with two doses and the VBI proportion of those with the heterologous booster BNT162b2. 

This particularity may be provoked by the time lapse between the booster dose administration and the occurrence of VBI (i.e., two to three months). During this period, the neutralizing activity against Omicron may have diminished, as reported in a recent study in persons who received a booster dose which found that their neutralizing activity was lost after three months [35]. However, even when the vaccine’s effectivity decreased over time, the effectivity against hospital admissions for COVID-19 remains high [29,36].

There is an association between the NAbs increment levels and the decrement in the risk of COVID-19 [37,38]. It has been observed that 99% of HCW with two doses of BNT162b2 had detectable titers of NAbs measured with Elecys 90 days after the second dose [39]; these were detectable up to 250 days after immunization, but there was a progressive decrease in their titers, mainly in those not previously infected [40]. Nevertheless, few studies have evaluated the NAbs’ protective threshold. Gilbert P. et al. [38] observed that patients with an inhibitory dilution of 50% (ID50), and NAbs with titers of 100 at day 57 post-vaccination, increased the vaccine’s efficacy from 50.8% to 90.7% in comparison with seronegative ones. Additionally, some studies have evaluated that the neutralizing titers were much lower against the Alpha and Delta variant, but principally against Omicron [32,41]. 

We found that the humoral response (NAbs evaluated with cPass™ that were greater than or equal to 60%, greater than or equal to 90%, and values greater than or equal to 500 employing the Elecsys^®^) 180 days after receiving the second BBIBP-CorV dose was associated with a lower risk of VBI. This phenom, evaluated during the third wave, suggested that a higher level of NAbs in the medium term (6 months) confers greater protection against COVID-19. This may serve as a future indicator of people’s susceptibility to SARS-CoV-2 infection. Nevertheless, we did not find any effect from the second dose after 210 days (which reflects the effectiveness of the BNT162b2 booster in most HWC). This was probably caused by the Nabs’ significant (and almost uniform) increment after this booster, even reaching above the cut-off points studied [4]. 

A previous SARS-CoV-2 infection at any moment between March and December 2021 was not associated with a lower risk of COVID-19 disease during the third wave. Studies have found that the immunological protection provoked by natural infection is heterogenic and changes according to certain factors such as age, sex, the severity of the disease, and the size of the initial inoculum, among others [42]. However, the protection period of these previous infections against reinfections by SARS-CoV-2 is 5 to 8 months [42,43]. This phenom might explain why no association was found between a history of previous SARS-CoV-2 infection in the present study and a lower risk of COVID-19 infection, as the time that had elapsed between previous infection and reinfection was longer than 12 months on average. It is also likely that these COVID-19 reinfections were due to the Omicron variant, which has a greater capacity for immune evasion [9,10]. 

We observed a significant increase in post-booster antibodies at the 210-day point after the second dose of BBIBP-CorV in those HCW who received a BNT162b2 booster (approximately 30 days prior). Additionally, antibody levels were higher in those previously infected in the four controls, either by cPass or by Elecys anti-SARS-CoV-2 S. However, a history of previous infection was not associated with protection against VBI, as detailed in Table 2. We did not observe differences in the levels of humoral response between those who developed the infection after the second dose or not, regardless of whether or not they received a booster. However, these data should be taken with caution due to the limited number of patients with COVID-19 infection after the second dose of BBIBP-CorV.

We observed that the male sex was a protective factor against VBI. When we internally compared the labor area according to gender, we observed that a large proportion of male HCW worked in administrative areas (49%). In comparison, most female HCW worked in customer service (42%) and analytical processes (36%), and only 0.3% worked in administrative areas. It is possible that due to remote work in administrative areas, male HCW have been less exposed to SARS-CoV-2.

This study has some limitations. Firstly, due to the study’s retrospective nature, it was impossible to evaluate variables that could influence the results, such as comorbidities and the severity of COVID-19. Secondly, only COVID-19 infection was considered the main outcome, and other important outcomes, such as hospitalizations or severity of the clinical condition, were not included. Furthermore, most participants were young, and we did not record comorbidities, which may be a risk factor for SARS-CoV-2 infection in HCW [44]. It was also not possible to include the occupational area variable in the regression analysis due to the small number of participants. Therefore, we cannot exclude that secondary to the higher percentage of male HCWs who worked in administrative areas (in remote work) have been less exposed to SARS-CoV-2, which may be a confounding factor in our findings. Finally, we did not evaluate the response mediated by cellular immunity, which has a fundamental role in preventing severe cases of COVID-19.

## 5. Conclusions

High levels of long-lasting antibodies (i.e., at 180 days) after a second dose of BBIBP-CorV may predict protection against VBI. We did not observe that a background of having received a heterologous booster with BNT162b2 or a history of previous SARS-CoV-2 infection was protective against VBI.

## Figures and Tables

**Table 1:** Demographics, characteristics and humoral response rates of the study population, and comparison between healthcare workers with and without a COVID-19 booster dose in vaccine breakthrough infection.

Variable	Total (*n* = 435)	VBI (*n*= 101)	Not VBI (*n* = 334)	*p*-Value
Demographic characteristics				
Age, years *	34.0 (28–42.5)	33 (27–38)	34 (29–43)	0.084 ^a^
Sex (%)				0.027 ^b^
Female	340 (78.16)	87 (86.14)	253 (75.75)	
Male	95 (21.84)	14 (13.86)	81 (24.25)	
Laboral area (%)				0.044
Phlebotomy	57 (13.10)	14 (13.86)	43 (12.87)	
Customer service	144 (33.10)	27 (26.73)	117 (35.03)	
Maintenance service	65 (14.95)	12 (11.89)	53 (15.87)	
Analytic process	122 (28.05)	40 (39.60)	82 (24.55)	
Administrative	47 (10.80)	8 (7.92)	39 (11.68)	
Infection before the third wave (%)	138 (31.72)	27 (26.73)	111 (33.23)	0.219 ^b^
Before the first dose of BBIBP-CorV	122 (28.04)	26 (25.74)	96 (28.74)	0.556 ^b^
After the first dose of BBIBP-CorV	11 (2.52)	0 (0.00)	11 (3.30)	0.075 ^c^
After the second dose of BBIBP-CorV	14 (3.21)	6 (5.94)	8 (2.40)	0.077 ^c^
Number of doses				0.633 ^b^
Two doses of BBIBP-CorV	138 (31.72)	34 (33.66)	104 (31.14)	
Two doses of BBIBP-CorV plus booster BNT162b2	297 (68.28)	67 (66.34)	230 (68.86)	
Time from the first infection until the third wave	507.5 (317–558)	541 (344–599)	492 (317–557)	0.084 ^a^
Days since first infection until VBI	-	539.5 (310–579)	-	
Days since infection before the first dose of BBIBP-CorV until VBI	-	555 (366–610)	-	
Days since infection after the second dose of BBIBP-CorV until VBI	-	175.5 (135–208)	-	
Humoral response rates21 days after the second dose (%)				
Elecys Anti-SARS-CoV-2 S (*n* = 303; VBI = 74, not VBI = 229) (+)				
≥0.8	296 (97.69)	73 (98.64)	223 (97.38)	0.999 ^c^
≥500	120 (39.60)	27 (36.48)	93 (40.61)	0.528 ^b^
≥1000	84 (27.72)	17 (22.97)	67 (29.25)	0.379 ^c^
Ab neutralization cPass (*n* = 280; VBI = 69, not VBI = 211) (+)				
≥30%	265 (94.64)	66 (95.65)	199 (94.31)	0.999 ^c^
≥60%	230 (82.14)	51 (73.91)	179 (84.83)	0.047 ^c^
≥90%	125 (44.64)	25 (36.23)	100 (47.39)	0.125 ^c^
90 days after the second dose (%)				
Elecys Anti-SARS-CoV-2 S (*n* = 384; VBI = 91, not VBI = 293) (+)				
≥0.8	372 (96.88)	86 (94.50)	286 (97.61)	0.165 ^c^
≥500	128 (33.33)	26 (28.57)	102 (34.81)	0.270 ^b^
≥1000	76 (19.79)	15 (16.48)	61 (20.81)	0.452 ^c^
Ab neutralization cPass (*n* = 356; VBI = 83, not VBI = 273) (+)				
≥30%	311 (87.36)	71 (85.54)	240 (87.91)	0.574 ^c^
≥60%	217 (60.96)	48 (57.83)	169 (61.90)	0.523 ^c^
≥90%	122 (34.27)	22 (24.17)	100 (36.63)	0.089 ^b^
180 days after the second dose (%)				
Elecys Anti-SARS-CoV-2 S (*n* = 395; VBI = 92, not VBI = 303) (+)				
≥0.8	384 (97.22)	88 (95.65)	296 (97.68)	0.290 ^c^
≥500	132 (33.42)	20 (21.73)	112 (36.96)	0.008 ^c^
≥1000	75 (18.99)	11 (11.95)	64 (21.12)	0.050 ^c^
Ab neutralization cPass (*n* = 383; VBI = 88, not VBI = 295) (+)				
≥30%	261 (68.15)	54 (61.36)	207 (70.16)	0.120 ^b^
≥60%	185 (48.30)	30 (34.09)	155 (52.54)	0.002 ^b^
≥90%	112 (29.24)	17 (19.31)	95 (32.20)	0.020 ^b^
210 days after second dose (%)				
Elecys Anti-SARS-CoV-2 S (*n* = 366; VBI = 91; not VBI = 275) (+)				
≥0.8	365 (99.73)	90 (98.90)	275 (100.0)	0.249 ^c^
≥500	323 (88.25)	78 (85.71)	245 (89.09)	0.452 ^c^
≥1000	307 (83.88)	75 (82.41)	232 (84.36)	0.742 ^c^
Ab neutralization cPass (*n* = 364; VBI = 90, not VBI = 274) (+)				
≥30%	345 (94.78)	84 (93.33)	261 (95.25)	0.584 ^c^
≥60%	329 (90.38)	80 (88.88)	249 (90.87)	0.544 ^c^
≥90%	307 (84.34)	76 (84.44)	231 (84.30)	0.999 ^c^

^a^: U Mann-Withney; ^b^: Chi^2^; ^c^: Fisher exact; * Median (interquartile range); (+) positive; VBI: vaccine breakthrough infection.

**Table 2:** Poisson regression analysis to evaluate predictors of COVID-19 vaccine breakthrough infection.

Variable	cRR (95% CI)	*p*-Value	aRR (95% CI)	*p*-Value
Male sex	0.575 (0.343–0.965)	0.037	0.430 (0.226–0.816)	0.010
Previously infected before third wave	0.785 (0.530–1.162)	0.227	1.688 (0.800–3.559)	0.169
Infection before the first dose of BBIBP-CorV	0.889 (0.599–1.319)	0.560		
Infection after the first dose of BBIBP-CorV	1.899 (1.010–3.569)	0.046		
Time since the first infection until the third wave	1.001 (0.998–1.004)	0.210		
Nº doses				
Two doses BBIBP-CorV	Ref.	-		
Two doses BBIBP-CorV plus booster BNT162b2	0.874 (0.612–1.247)	0.459		
Ab neutralization cPass at 21 days ≥ 60%	0.615 (0.395–0.958)	0.032	0.621 (0.397–0.971)	0.037
Ab neutralization cPass at 180 days ≥ 60%	0.553 (0.373–0. 820)	0.003	0.588 (0.396–0.874)	0.009
Ab neutralization cPass at 180 days ≥ 90%	0.579 (0.357–0.938)	0.026	0.598 (0.371–0.964)	0.035
Elecys Anti-SARS-CoV-2 S at 180 days ≥ 500	0.553 (0.353–0.867)	0.010	0.585 (0.373–0.916)	0.019

cRR: crude relative risk; aRR: adjusted relative risk. BBIBP-CorV: inactivated vaccine against SARS-CoV-2 BBIBP-CorV (Sinopharm). BNT162b2: Vaccine ARNm BNT162b2 (Pfizer/BioNTech). The Variables: Ab neutralization cPass at 21 days ≥ 60%, Ab neutralization cPass at 180 days ≥ 60%, Ab neutralization cPass at 180 days ≥ 90% and Elecys anti-SARS-CoV-2 S at 180 days ≥ 500; since they were not independent of each other, they were analyzed individually. The variables were adjusted by sex.

## Data Availability

The data analyzed in this manuscript, as well as their definitions, can be downloaded at the DOI: 10.17632/3rx5mhntmp.1.
